# Characterization of Retinal Microvascular Abnormalities in Birdshot Chorioretinopathy Using OCT Angiography

**DOI:** 10.1016/j.xops.2024.100559

**Published:** 2024-06-17

**Authors:** Aman Kumar, Alexander Zeleny, Sunil Bellur, Natasha Kesav, Enny Oyeniran, Kübra Gul Olke, Susan Vitale, Wijak Kongwattananon, H. Nida Sen, Shilpa Kodati

**Affiliations:** 1 National Eye Institute, National Institutes of Health, Bethesda, Maryland; 2 Kellogg Eye Center, Department of Ophthalmology, University of Michigan

**Keywords:** Birdshot chorioretinopathy, Optical coherence tomography angiography, Retinal vessel density, Uveitis

## Abstract

**Objective:**

To characterize changes in the retinal microvasculature in eyes with birdshot chorioretinopathy (BCR) using OCT angiography (OCTA).

**Design:**

Retrospective, observational, single center.

**Subjects:**

Twenty-eight patients (53 eyes) with BCR and 59 age-matched controls (110 eyes).

**Methods:**

En face OCTA images of the superficial capillary plexus (SCP) and deep capillary plexus (DCP) of each eye were assessed for the presence of microvascular abnormalities and used to measure the vessel and foveal avascular zone (FAZ) areas. A longitudinal analysis was performed with a representative cohort of 23 BCR eyes (16 patients) at baseline and at a 2-year time point.

**Main Outcome Measures:**

Whole-image vessel density (VD, %), extrafoveal avascular zone (extra-FAZ) VD (%), and FAZ area (%) were calculated and compared between control and BCR eyes. The frequency of microvascular abnormalities in BCR eyes was recorded.

**Results:**

In the SCP, increased intercapillary space and capillary loops were common features present on OCTA images. Whole-image and extra-FAZ VD were lower in the BCR group compared with controls (*P* < 0.0001 [SCP and DCP]). Foveal avascular zone area was enlarged in BCR eyes (*P* = 0.0008 [DCP]). Worsening best-corrected visual acuity was associated with a decrease in whole-image and extra-FAZ VD in the SCP (*P* < 0.0001 for both) and the DCP (*P* < 0.005 for both). Multivariable analysis, with vessel analysis parameters as outcomes, demonstrated that increasing age, increasing disease duration, lower central subfield thickness, and treatment-naive eyes (compared with those on only biologics) were associated with a significant decrease in both DCP whole-image and extra-FAZ VD. Increasing disease duration was associated with a significant decrease in both SCP whole-image and extra-FAZ VD. Longitudinal analysis demonstrated no significant difference in any vessel analysis parameters except for an increase in DCP FAZ area.

**Conclusions:**

Our results demonstrate a significant a decrease in VD in BCR eyes and an association on multivariable analysis with disease duration. Quantifying VD in the retinal microvasculature may be a useful biomarker for monitoring disease severity and progression in patients with BCR. Further studies with extended longitudinal follow-up are needed to characterize its utility in monitoring disease progression and treatment response.

**Financial Disclosure(s):**

Proprietary or commercial disclosure may be found in the Footnotes and Disclosures at the end of this article.

Birdshot chorioretinopathy (BCR) is a chronic posterior uveitis characterized by cream-colored choroidal lesions, retinal vasculitis, and HLA-A29 positivity.^1^, ^2^, ^3^ The disease BCR has a female preponderance, is more common in those with northern European ancestry, and has an estimated prevalence of 0.2 to 1.7 cases per 100 000.^4^

In BCR, inflammatory changes in the retina and choroid may occur independently.^5^, ^6^, ^7^ Therefore, multimodal imaging techniques including OCT, fluorescein angiography (FA), and indocyanine green angiography are important in the initial diagnosis and monitoring of disease activity.^8^, ^9^, ^10^, ^11^ OCT angiography (OCTA) is a noninvasive modality that permits a detailed en face view of the vascular layers including the superficial and deep retinal capillary plexi using a motion contrast algorithm to detect flow produced by erythrocyte movement.^12^, ^13^, ^14^ OCT angiography permits identification of subtle microvascular abnormalities that are not detectable by FA, visualization of the distinct capillary plexi, and allows for quantification of the retinal microvasculature.^12^^,^^15^^,^^16^ OCT angiography has demonstrated utility in identifying flow abnormalities in the retinal vasculature in patients with posterior uveitis.^17^

Previously, we reported the repeatability of quantitative metrics derived from OCTA images in a cohort of patients with posterior segment uveitic diseases. We found reliable intravisit repeatability in retinal vascular parameters, including with superficial capillary plexus (SCP) and deep capillary plexus (DCP) vessel density (VD), and foveal avascular zone (FAZ) measurements in our series.^18^ Recent studies have described increased intercapillary spacing (ICS), increased capillary dilations and loops, and increased telangiectatic vessels in BCR eyes compared with control eyes.^19^, ^20^, ^21^, ^22^ However, there remains a paucity of data on the utility of quantitative features of OCTA in BCR. Changes in OCTA parameters observed in BCR may indicate disruption of the microvascular circulation in the macula and reflect disease severity. Thus, OCTA parameters may potentially serve as biomarkers for monitoring the progression and prognosis of the disease. The aim of this study was to utilize OCTA to investigate the changes in the retinal microvasculature compared with healthy controls, and their association with parameters such as visual acuity and disease duration for the purpose of better understanding the pathophysiology of BCR and determining biomarkers for disease progression.

## Methods

### Study Design and Participants

This study was a retrospective single-center study conducted at the uveitis clinic at the National Eye Institute, National Institutes of Health, Bethesda, Maryland, United States from December 2016 to June 2021. The subjects included both healthy controls and those diagnosed with BCR.^23^ The study received approval by the National Institutes of Health Institutional Review Board and adhered to the tenets of the Declaration of Helsinki. Due to the retrospective nature of this study, the requirement for informed consent was waived. All participants with BCR were HLA-A29 positive. Fundus imaging, FA, indocyanine green angiography, and OCT imaging were previously performed on all patients to support the diagnosis.

### Data Collection

Information on demographics, comorbidities, ocular history, clinical findings, ancillary examinations, and treatment was extracted from the electronic medical record. Eyes were clinically active if they had clinical examination or imaging findings concerning for disease activity including ≥1 of the following: new onset or increase in vitreous haze, increased retinal vascular leakage, and/or disc leakage on fundus fluorescein angiogram. Eyes were deemed clinically quiet if they were without any evidence suggestive of active inflammation (clinical findings and/or fundus fluorescein angiogram).^24^ Demographic data and clinical findings included age, sex, ethnicity, best-corrected visual acuity (BCVA), refractive error, central subfield thickness (CST), ocular injections, systemic corticosteroid therapy (prednisone), conventional immunomodulatory therapy (mycophenolate mofetil, cyclosporine, tacrolimus), and biologics (infliximab and adalimumab). Disease duration was determined from the date of diagnosis to the date of the acquired image.

### OCTA Image Acquisition Protocol and Analysis

OCT angiography images from eyes with BCR were obtained at the earliest available time point. Both eyes of participants were included for our final analysis unless an eye or image met the following exclusion criteria: (1) OCTA image with signal strength <7 out of 10, (2) substantial motion artifact or segmentation error, (3) coexistent retinal and choroidal diseases other than uveitis including age-related macular degeneration or central serous chorioretinopathy, (4) previous history of macular laser, and (5) refractive errors greater than +6.0 or −6.0 diopters.

We obtained 6 × 6 mm^2^ OCTA scans centered on the fovea using the CIRRUS AngioPlex Model 5000 (Carl Zeiss Meditec). Automated segmentation of the SCP and DCP were used. The SCP extended from the inner limiting membrane to the inner plexiform layer and the DCP extended from the inner nuclear layer to the outer plexiform layer. These slabs were used further for quantitative analysis of the flow and vasculature.^25^ Images were then exported into ImageJ (version 1.51g, National Institutes of Health) for image preprocessing and thresholding analysis.

**Figure 1:**
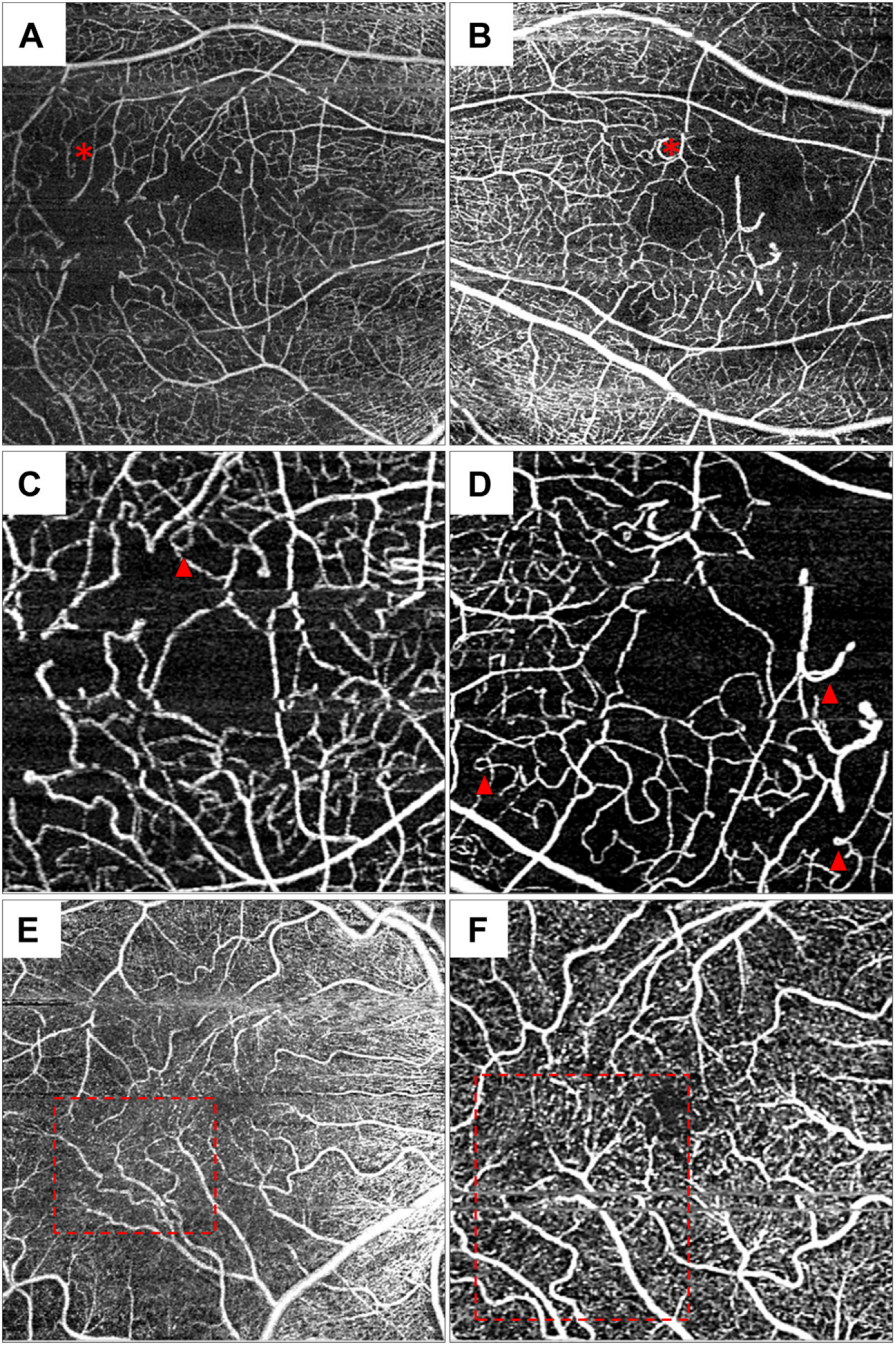
Bilateral (**A, B**) 6 × 6 mm^2^ and (**C, D**) 3 × 3 mm^2^ en face OCT angiography images demonstrating decreased vessel density. Findings included increased capillary spacing (asterisk), capillary loops (red arrowhead), and (**E, F**) decreased vessel density with areas of vascular tortuosity (red, dashed rectangle) in the superficial capillary plexus.

Qualitative analysis of 3 × 3 mm^2^ and 6 × 6 mm^2^ en face images on the Cirrus HD-OCT system were independently performed by 2 graders (E.O. and S.B.). For any cases of discrepancies, a consensus grading was performed by an adjudicator (S.K.). All images were evaluated in clinic in a similar environment. Each image was evaluated for the presence or absence of several qualitative metrics in the SCP and DCP: capillary loops, tortuous vessels, increased ICS, changes in VD, and either loss of vasculature or inability to distinguish the superficial and deep capillary plexi (Fig 1).^20^ For the last-mentioned, graders assessed images by scrolling through the OCT slab images on the OCTA system.

### OCTA Image Processing and Analysis

OCT angiography images from both patients with BCR and healthy, age-matched controls evaluated at our institution underwent quantitative analysis. A semiautomatic algorithm was utilized to quantify VD and FAZ area in the SCP and DCP. Images were standardized by brightness histogram contrast stretching. Each 6 × 6 mm^2^ image was imported into ImageJ where a customized macro selected a central square at 50% of the image size to account for variable brightness in the peripheries as previously described.^26^ Intensity values for the whole-image were automatically normalized to produce a standardized brightness histogram.^27^

**Figure 2:**
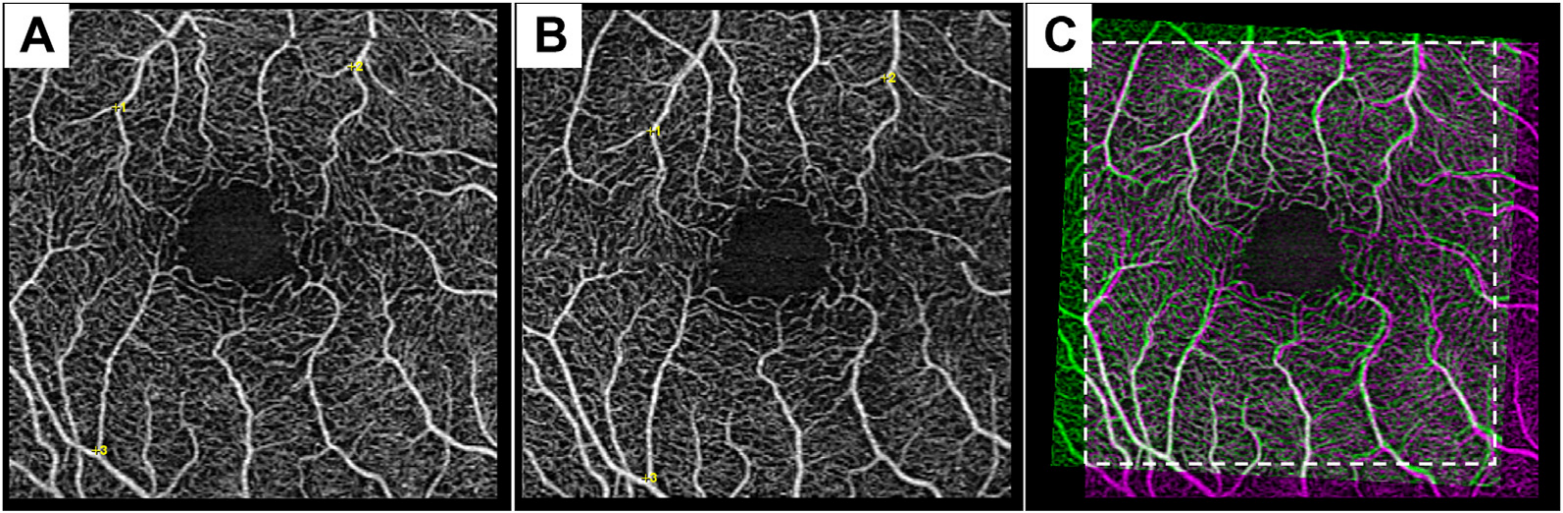
Alignment of 2 images of the same eye over a 2-year period. **A, B,** En face OCT angiography of the superficial capillary plexus with 3 points at vessel junctions (yellow). **C,** Overlapping areas are white (**A**, magenta) and (**B**, green) images. A white bounding box indicates the area use for intervisit comparison.

For the longitudinal component of our analysis, an additional step was taken prior to threshold analysis. Images across 2 visits for the same patient were aligned using feature-based image registration.^28^ Processed SCP and DCP images of the same eye at the 2-year time point and baseline were imported into ImageJ and automatically registered using the similarity method on TrakEM which preserves the image aspect ratio (Fig 2). These images were reviewed for any significant discrepancies and the overlapping area between all visits were manually cropped and exported for threshold analysis.

**Figure 3:**
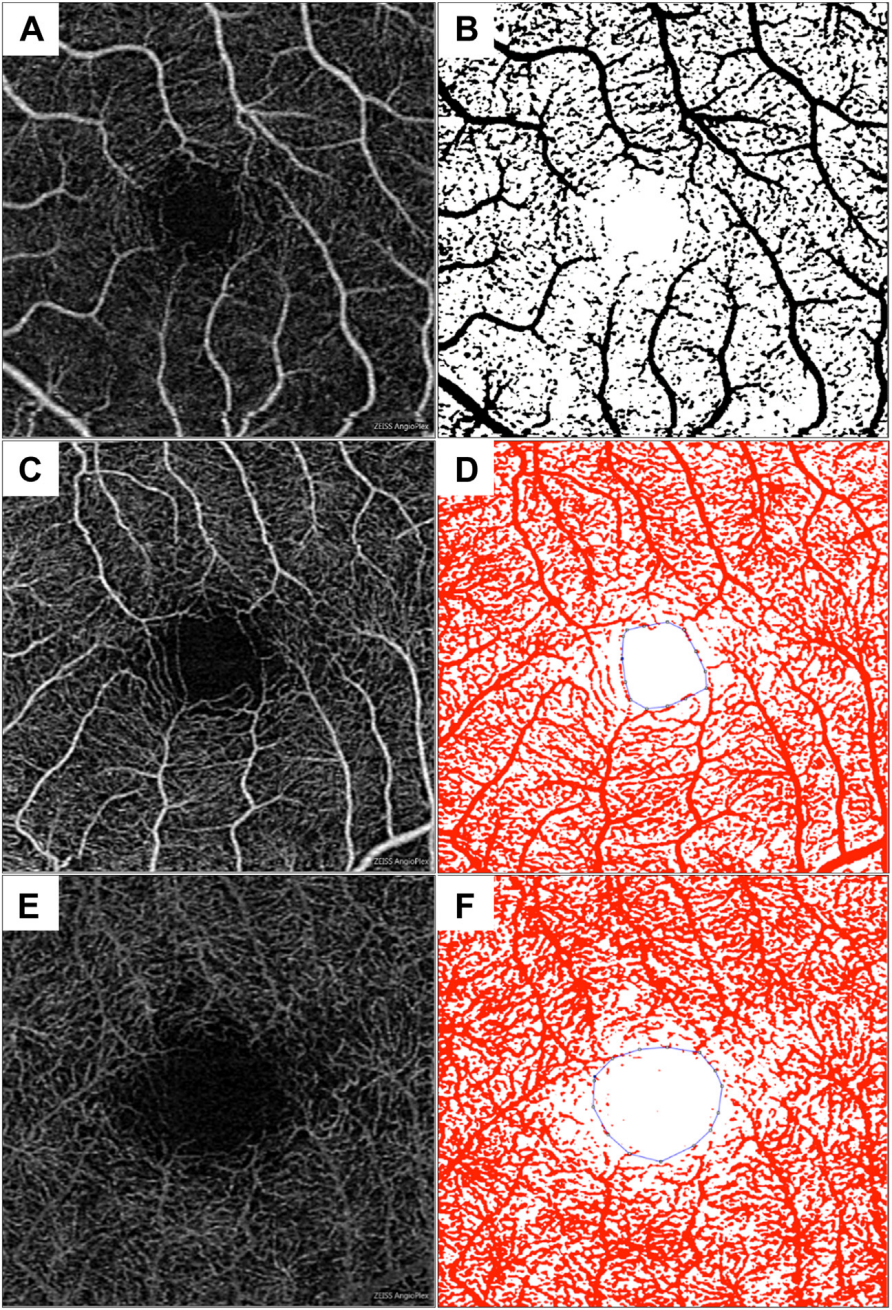
Image analysis pipeline. (**A**) and (**B**) demonstrate en face OCT angiography (OCTA) images of the superficial capillary plexus (SCP) and its respective binarized image. (**C**) and (**D**) represent en face OCTA images of the SCP and the grader’s outlining of the SCP foveal avascular zone area. (**E**) and (**F**) represent en face OCTA images of the deep capillary plexus (DCP) and the outlining of the DCP foveal avascular zone area. The area in red was quantified as the vessel area for both the SCP (**D**) and DCP (**F**).

As previously described, the standardized images of the SCP and DCP were then binarized in ImageJ by local Otsu thresholding to demonstrate the vessel area in black pixels and nonvessel areas of the OCTA as a white background.^29^ Using the “measure” option, the vessel area was then calculated (Fig 3A, B).^26^ To calculate the FAZ area, the FAZ in the SCP and DCP were first manually outlined by the trained user using the polygon tool (Fig 3C–F). Then, using the “measure” option again, the area index of the layers was computed. The values for vessel area and FAZ area were exported to calculate the VD. In this study, the term “VD” refers to the vessel area divided by the image area: whole-image VD (vessel area divided by the total image area) and the extrafoveal avascular zone (extra-FAZ) VD (vessel area divided by the total image area—FAZ area).

### Multimodal Imaging

Multimodal imaging evaluation was performed by 2 fellowship-trained uveitis specialists (S.B. and E.O.). Fluorescein angiography was graded based on the late frames, with attention to the presence or absence of optic disc leakage, macula leakage, and retinal vascular staining/leakage. Indocyanine green angiography Spectralis HRA was based on review of early (<3 minutes), middle (10 minutes), and late phases (28–42 minutes) with focus on the presence or absence of hypofluorescent lesions, fuzzy, indistinct choroidal vessels, atrophic areas, and late-diffuse choroidal hypofluorescence. OCTs were graded and assessed for the presence of a disrupted external limiting membrane, ellipsoid zone, and retinal pigment epithelium/Bruch’s membrane complex.^8^

### Statistical Methods

Statistical analysis was performed using GraphPad Prism version 9.1.2 (GraphPad Software Inc) for all analyses except for longitudinal comparison and multivariable analyses, for which SAS (version 9.4, SAS Institute) was utilized. All the analyses that were performed at eye level had appropriate statistical adjustments made for the correlation between eyes of an individual. Chi-squared tests were used to compare percentages between groups. At baseline, Mann–Whitney tests were used to compare control and BCR groups for each vessel analysis parameter. A clustered Wilcoxon signed rank test was performed to assess longitudinal changes in parameters between baseline and the 2-year time point, adjusting for correlation between eyes of an individual. Pearson correlation coefficients were used to measure the relationship between vessel analysis parameters and covariates. We generated separate linear regression models (SAS Proc GENMOD) for each outcome variable of interest (the 6 angiographic parameters and BCVA). For each outcome variable, we first computed univariable models for each covariate (age, sex, CST, estimated disease duration [months], history of epiretinal membrane [yes/no]), current oral agents (T-cell inhibitors/antimetabolite), current biologics (yes/no), current oral prednisone use (yes/no), and clinical disease activity (active/quiet). Covariates found to be nominally associated with the angiographic parameters in the univariable analysis (*P* < 0.10) served as independent variables in subsequent multivariable analysis. All *P* values were 2-sided and considered statistically significant if their values were *P* < 0.05.

## Results

### Characterizing Microvascular Changes in BCR

A total of 53 eyes from 28 patients with BCR and 110 eyes from 59 control subjects were included in this analysis. Age was similar between the BCR group (60.04 ± 11.0 years) and control group (58.15 ± 16.2 years; *P* = 0.774). Best-corrected visual acuity (logarithm of the minimum angle of resolution [logMAR]) was better in the control group (0.0417 ± 0.0987 [20/20]) than the BCR group (0.0717 ± 0.141 [20/23]; *P* = 0.0318). Central subfield thickness was significantly higher in the control group (262 ± 20.8 μm) compared with the BCR group (254 ± 59.9 μm; *P* = 0.0377). These data are summarized in Table 1. The mean estimated disease duration was 5.71 ± 5.51 years. A total of 31 (58.49%) eyes were clinically active. A total of 15.09% (8 eyes) had the presence of intraretinal fluid and 13.21% (7 eyes) were noted to have disorganization of the retinal inner layers. Additional baseline characteristics are summarized in Table 2.

**Table 1:** Cross-Sectional Baseline Comparison of BCR and Control Groups

Variable	BCR	Control	*P* Value
Patients	28	59	
Baseline age, years (mean ± SD)	60.04 ± 11.0	58.15 ± 16.2	0.774
Sex, female, n	19	32	0.253
Eyes	53	110	
Best-corrected visual acuity, logMar (mean ± SD)	0.0717 ± 0.141	0.0417 ± 0.0987	0.0318
Central subfield thickness, μm (mean ± SD)	254 ± 59.9	262 ± 20.8	0.0377

BCR = birdshot chorioretinopathy; logMAR = logarithm of the minimum angle of resolution; SD = standard deviation.

**Table 2:** Birdshot Chorioretinitis Demographic Data and Clinical Findings

Variable (or Characteristic)	Value
Spherical equivalent (mean ± SD)	−0.70 ± 2.34
Disease duration, years (mean ± SD)	5.71 ± 5.51
Disease activity; n (% active)	31 (58.49%)
Current treatment; n (%)	
Injection/Ozurdex last 6 months; Retisert in last 3 years	6 (11.32%)
Oral prednisone	3 (10.71%)
Oral prednisone (≥10 mg)	2 (7.14%)
Oral immunomodulatory treatment (antimetabolites/T-cell inhibitors)	18 (64.29%)
Biologics	10 (35.71%)
Ocular surgery; n (%)	16 (30.19%)
Cataract severity; n (%)	
None	22 (41.51%)
<2+	12 (22.64%)
>2+	2 (3.77%)
Pseudophakic	17 (32.08%)
Anterior chamber cell; n (%)	
None	52 (98.11%)
Trace	0 (0%)
1+	1 (1.89%)
≥2+	0 (0%)
Vitreous cell; n (%)	
None	34 (64.15%)
Trace	10 (18.87%)
1+	9 (16.98%)
≥2+	0 (0%)
Vitreous haze; n (%)	
None	50 (94.34%)
Trace	2 (3.77%)
1+	1 (1.89%)
≥2+	0 (0%)
CME (current); n (% eyes)	6 (11.32%)
Past ocular history; n (%)	
CME	12 (22.64%)
Epiretinal membrane	23 (43.64%)
Choroidal neovascularization	2 (3.77%)
Diabetic retinopathy	0 (0%)
Age-related macular degeneration	0 (0%)
Vein occlusions	1 (1.89%)
Glaucoma	8 (15.09%)

CME = cystoid macular edema; SD = standard deviation.

#### Qualitative Analysis

For the qualitative analysis, FA and ICG were obtained within 3 months of the initial OCTA. Fluorescein angiography was available in 32 of 53 eyes (62.38%) and indocyanine green angiography was recorded in 30 of 53 eyes (56.60%). A summary of the multimodal imaging findings are available in Table 3.

**Table 3:** Disease Activity Scoring Utilizing FA and ICGA

Multimodal Imaging Findings	% (Number of Eyes)
Disc leakage on FA	75.0% (24)
Retinal vascular leakage	75.0% (24)
Macular leakage on FA	46.9% (15)
Atrophic lesions on ICG	56.2% (18)
Active lesions on ICG	76.7% (23)
Disruption of ELM, EZ, and RPE/BM complex on OCT	6.2% (2)

ELM = external limiting membrane; EZ = ellipsoid zone; FA = fluorescence angiography; ICG = indocyanine green; ICGA = indocyanine green angiography; RPE/BM = retinal pigment epithelium/basement membrane.

Analysis of the microvasculature on OCTA in the SCP and DCP were qualitatively assessed for abnormal findings. In the SCP, decreased VD (32.1%), increased ICS (30.2%), and capillary loops (26.4%) were relatively commonly seen. Tortuous vessels were less common (9.43%). In the DCP, nearly half of the eyes (41.5%) demonstrated decreased VD. Infrequently, the lack of a distinct DCP was observed (i.e., the inability to distinguish SCP/DCP from each other (7.55%). These data are summarized in Table 4.

**Table 4:** Summary of Retinal and Choroidal Microvasculature Alterations Observed in Macular 6 × 6 mm^2^ En Face OCTA Images of Eyes With Birdshot Chorioretinopathy

OCTA Grading Parameters (%)	% (Number of Eyes)
Decreased vessel density (SCP)	32.1% (17)
Decreased vessel density (DCP)	41.5% (22)
Capillary loops (SCP)	26.4% (14)
Tortuous vessels (SCP)	9.43% (5)
Increased intercapillary space (SCP)	30.2% (16)
(1) Presence/absence of loss of either SCP/DCP or (2) inability to distinguish SCP/DCP from each other	7.55% (4)

DCP = deep capillary plexus; OCTA = OCT angiography; SCP = superficial capillary plexus.

#### Quantitative Analysis

**Figure 4:**
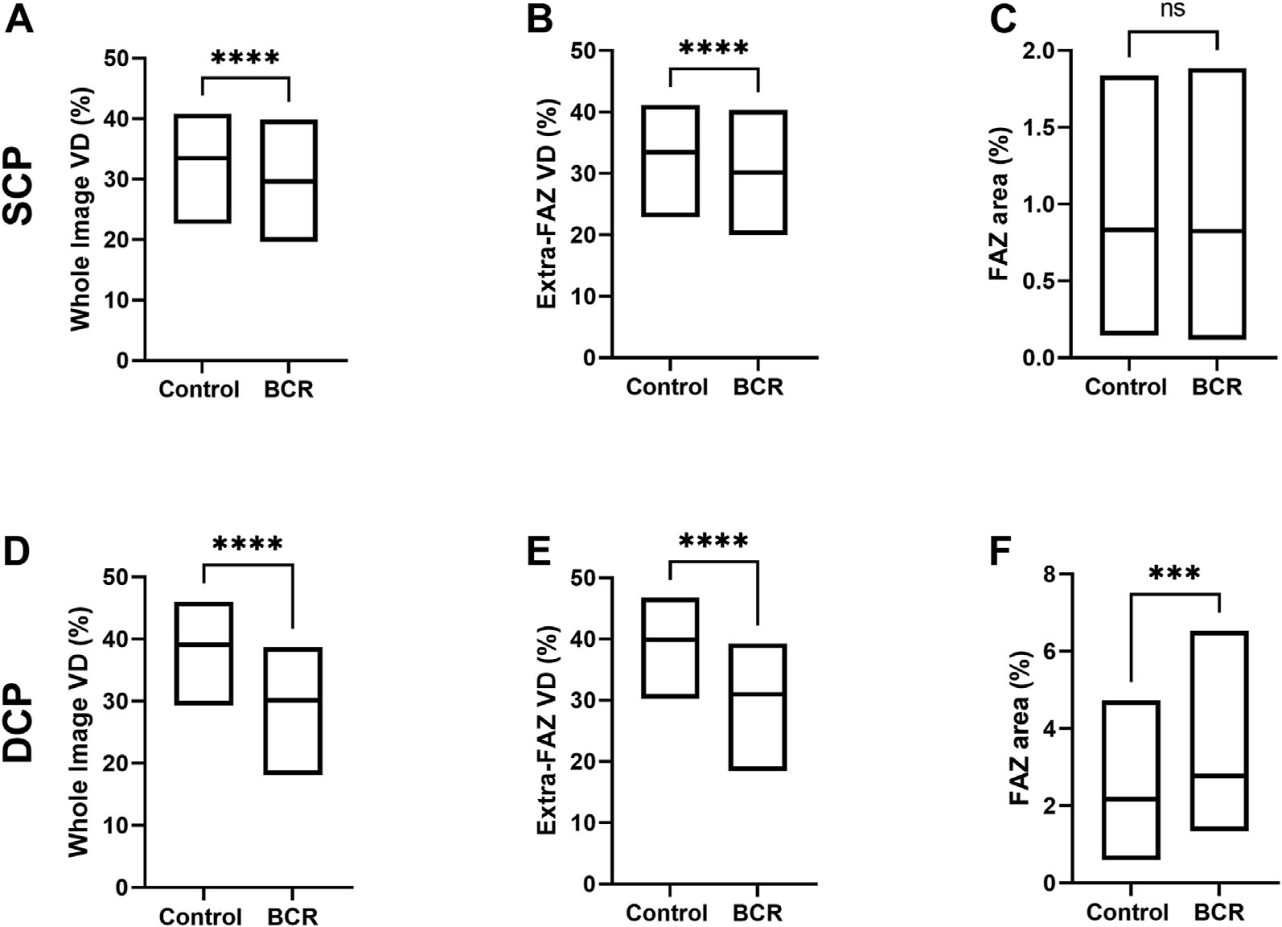
Boxplots demonstrating differences in means between the BCR and control groups for vessel analysis parameters: whole-image VD, extra-FAZe VD, and FAZ area in the SCP (**A**–**C**) and the DCP (**D**–**F**). BCR = birdshot chorioretinopathy; DCP = deep capillary plexus; extra-FAZ = extrafoveal avascular zone; FAZ = foveal avascular zone; SCP = superficial capillary plexus; VD = vessel density. ∗∗∗*P* < 0.001, ∗∗∗∗*P* < 0.0001.

In the SCP, whole-image VD and extra-FAZ VD were significantly lower in the BCR group (29.9 ± 4.80; 30.1 ± 4.78) than in the control group (33.2 ± 4.40 [*P* < 0.0001]; 33.4 ± 4.43 [*P* < 0.0001]). Foveal avascular zone area was similar in the control and BCR groups (0.83 ± 0.35; 0.83 ± 0.41; respectively, *P* = 0.765) (Fig 4A–C). In the DCP, whole-image VD and extra-FAZ VD were also significantly lower in the BCR group (30.0 ± 6.16; 30.9 ± 6.22) than in the control group (39.1 ± 3.45 [*P* < 0.0001]; 39.9 ± 3.50 [*P* < 0.0001]). Foveal avascular zone area was lower in the control group (2.17 ± 0.75) compared with the BCR group (2.77 ± 1.12; *P* = 0.0008) (Fig 4D–F).

### Vessel Analysis Parameters and Visual Acuity

In the SCP, increasing logMAR (Fig S5A–C, available at www.ophthalmologyscience.org) was significantly associated with lower whole-image VD (r = −0.523, *P* < 0.0001) and extrafoveal VD (r = −0.530, *P* < 0.0001). In the DCP, increasing logMAR (Fig S5D–F, available at www.ophthalmologyscience.org) was also significantly associated with lower whole-image VD (r = −0.383, *P* = 0.00460) and extrafoveal VD (r = −0.381, *P* = 0.00494). Throughout the analysis an outlier was present and was included in our analysis because it held clinical relevance after thorough clinical review. After outlier removal, correlations with vessel analysis parameters remained significant. No significant correlation was seen with SCP or DCP FAZ area and BCVA. In the SCP, increasing logMAR (Fig S5A–C, available at www.ophthalmologyscience.org) was weakly associated with increasing FAZ area (r = 0.0346, *P* = 0.806). In the DCP, increasing logMAR (Fig S5D–F, available at www.ophthalmologyscience.org) was associated with increasing FAZ area (r = 0.203, *P* = 0.145).

### Multivariable Analysis of Factors Associated With Vessel Analysis Parameters

Variables examined by using univariable analyses included age, sex, CST, estimated disease duration (months), history of epiretinal membrane (yes/no), current oral agents (T-cell inhibitors/antimetabolite), current biologics (yes/no), current oral prednisone use (yes/no), and clinical disease activity (active/quiet). Sex, history of epiretinal membrane, current oral prednisone use, and clinical disease activity were not significantly associated with any of the vessel analysis parameters (whole-image VD, extra-FAZ VD, and FAZ area) in the univariable models and were not considered further. The remaining variables were included in the final multivariable model for each parameter, summarized in Table S5 (available at www.ophthalmologyscience.org). With older age, DCP whole-image and extra-FAZ VD decreased (−0.31 [*P* < 0.0001]; −0.31 [*P* < 0.0001]) and SCP and DCP FAZ area increased (0.011 [*P* = 0.0038]; 0.050 [*P* = 0.011]). Decreasing CST was associated with increasing DCP whole-image and extra-FAZ VD (0.027 [*P* < 0.0001]; 0.026 [*P* < 0.0001]) and increased SCP and DCP FAZ area (−0.0020 [*P* = 0.036]; −0.0053 [*P* < 0.0001]). In the SCP, increased duration of disease was significantly associated with decreased whole-image VD (−0.16 [*P* = 0.0069]), extra-FAZ VD (−0.16 [*P* = 0.0055]), and (borderline association) SCP FAZ area (−0.0099 [*P* = 0.11]). In the DCP, increased disease duration was significantly associated with decreased whole-image VD (−0.18 [*P* < 0.0001], extra-FAZ VD (−0.11 [*P* < 0.0001]), and (borderline association) decrease in FAZ area (−0.026 [*P* = 0.089]). To assess possible effects of different treatments on these parameters, current biologics was used as the reference category and was compared against the other 3 treatment regimens: no current treatment, current oral agents, and dual therapy (biologics and oral agents together). Deep capillary plexus whole-image and extra-FAZ VD were decreased (−7.42 [*P* = 0.0001]; −7.39 [*P* = 0.0002]) and DCP FAZ area was increased (0.73 [*P* = 0.022]) in treatment-naive eyes compared with those on only biologics. However, there was no significant difference in any parameter when biologics alone were compared with oral agents or with dual therapy.

### Longitudinal Analysis

We obtained longitudinal data at 2 years on 23 eyes of 16 patients. When compared with the cross-sectional group, the longitudinal cohort did not differ with respect to age, sex, or CST. The average age was 59.3 ± 8.40 years. Most patients were female (11/16). We compared changes from baseline to 2 years. Best-corrected visual acuity (logMAR) did not change significantly over time (2-year, 0.049 ± 0.121; baseline, 0.0458 ± 0.0833 [*P* = 1.0]. Central subfield thickness (μm) was slightly lower at 2 years (236 ± 47.0) compared with baseline (239 ± 32.2 [*P* = 0.966]). Clinical disease activity either improved (n = 11 eyes) or remained inactive (n = 10 eyes), except for 2 eyes from 1 patient, which remained active. This patient continued oral therapy and received an oral prednisone taper as the sole additional treatment.

In the SCP, whole-image VD, extra-FAZ VD, and FAZ area after 2 years (30.5 ± 5.45; 30.8 ± 5.49; 0.977 ± 0.40) remained similar to baseline (31.0 ± 5.21 [*P* = 0.730]; 31.2 ± 5.25 [*P* = 0.730]; 0.962 ± 0.42 [*P* = 0.463]). In the DCP, whole-image and extra-FAZ VD at 2 years (30.7 ± 5.26; 31.7 ± 5.38) remained stable compared with baseline (31.19 ± 5.93 [*P* = 0.641]; 32.1 ± 5.98 [*P* = 0.219]). However, FAZ area was significantly higher at 2 years (3.18 ± 0.52) than at baseline (2.87 ± 0.66 [*P* = 0.0304]).

## Discussion

In this study, we characterized microvascular changes in a cohort of patients with BCR. Both qualitative and quantitative microvascular changes were observed on OCTA. To the best of our knowledge, our quantitative analysis represents the largest age-matched comparison of BCR to controls to date. To summarize, our results demonstrate (1) qualitative microvascular abnormalities in BCR including the inability to detect a distinct DCP in advanced disease, (2) significantly reduced SCP and DCP VD in BCR compared with healthy controls, (3) the association of decreased VD with decreasing BCVA consistent with previous studies, (4) an inverse association of VD with disease duration, and (5) an absence of longitudinal progression in decreased VD within a 2-year period.

Previous studies have conducted cross-sectional, retrospective analyses reporting qualitative OCTA findings in eyes with BCR. They have observed the presence of capillary loops, telangiectasis, and increased ICS throughout the posterior pole.^8^^,^^10^^,^^19^^,^^20^^,^^22^^,^^30^ These studies have indicated that the frequency of these abnormalities may be related to disease activity^8^^,^^10^ and disease duration.^8^ In our study, eyes with clinically active disease (n = 31) demonstrated a lower frequency of all retinal microvascular abnormalities compared with inactive eyes (n = 22) except for increased ICS in the SCP which was lower in active eyes (*P* = 0.041, chi-squared). However, the disease duration in the active eyes was lower (4.17 years) compared with inactive eyes (7.87 years; *P* = 0.018) suggesting that these changes may accumulate over time in the retinal vasculature and that disease duration may be an important factor in the development of these microvascular changes. In the retina, reduced VD, increased vessel tortuosity, and collapse may be associated with hypoxia.^8^^,^^19^^,^^31^^,^^32^ Increased tortuosity is thought to result from arteriolar dilation due to tissue hypoxia, and may be driven by the release of angiogenesis-promoting mediators to improve tissue perfusion.^33^, ^34^, ^35^, ^36^ Moreover, increased vessel tortuosity may also indicate underlying vessel wall dysfunction and blood-retinal barrier damage.^31^^,^^37^^,^^38^

We observed a significant quantitative reduction in VD of both the SCP and DCP compared with age-matched controls. We also found that decreasing whole-image and extra-FAZ VD in the SCP and DCP correlated with decreased BCVA. Prior studies have noted similar reductions in VD in both SCP and DCP in autoimmune posterior uveitis and posterior uveitis compared with healthy controls.^39^ Roberts et al quantified 3 × 3 mm^2^ OCTA images to measure retinal VD and FAZ area in 37 eyes of 21 patients with BCR compared with age-matched controls.^21^^,^ They reported significantly decreased VD in the SCP and DCP of patients with BCR and an association with visual acuity.

Notably, the changes in VD seen in BCR were apparent even when SCP and DCP whole-image VD for controls and BCR were graphed against age (Fig S6B, available at www.ophthalmologyscience.org). However, despite observing evidence for both SCP and DCP involvement, our results suggest a greater degree of VD reduction in the DCP in BCR (Fig S6B–D, available at www.ophthalmologyscience.org). A disproportional loss of VD in the DCP has been previously described in several other conditions: retinal vessel occlusion,^40^, ^41^ smoking,^42^ diabetic retinopathy,^43^, ^44^ Vogt–Koyanagi–Harada disease,^45^ and Behḉet's posterior uveitis.^46^, ^47^ A case study described diminished DCP VD in a patient with BCR complicated by neovascularization when compared with controls.^48^ Repetitive vascular inflammatory insult in combination with the distal nature of the DCP (oxygen desaturation and reduced perfusion pressure) may make the DCP more susceptible to hypoxia,^49^^,^ which can promote oxidative stress.^42^^,^^50^, ^51^, ^52^ Overtime, this could result in endothelial dysfunction, vascular remodeling, and capillary dropout in the outer retinal layers. Interestingly, a distinct DCP could not be clearly visualized in 4 eyes with advanced disease in our study and corresponding BCVA ranged from 0.1 to 0.9 logMAR. Previous studies have also demonstrated that deep retinal capillary nonperfusion may be related to photoreceptor dysfunction. Specifically, the DCP is essential in supplying the metabolic needs of the outer plexiform layer^53^ and decreased perfusion of the DCP may result in photoreceptor compromise. Spectral domain-OCT has revealed the macular thinning in BCR to be predominantly in the outer retina.^4^^,^^10^^,^^54^^,^^55^ Additionally, significantly reduced cone density on adaptive optics in eyes with BCR compared with controls has been previously reported.^20^ However, despite this evidence, it remains unclear whether the changes observed in VD in BCR are a cause of photoreceptor dysfunction or a reflection of the disease process.^21^ Our multivariable analysis showed that increasing disease duration was significantly associated with a decrease in whole-image and extra-FAZ VD in both the SCP and DCP, highlighting the chronic, progressive disease course of BCR.

Previous studies have noted the SCP and DCP as potential treatment markers in posterior uveitis. Wassef et al conducted a prospective longitudinal observational analytic study lasting on average 122.68 ± 84.25 days in which patients with active Behḉet's posterior uveitis underwent OCTA imaging during activity and after remission demonstrated increases in the VD in both the SCP and DCP.^56^ We investigated changes in VD over a 2-year period in a subgroup of our patients and observed minimal change in both SCP and DCP VD. The observed stability in VD over a span of 2 years implies that this timeframe may be insufficient for discerning significant progression. However, we did observe significant enlargement of the DCP FAZ area over 2 years, although it remains unclear whether this change is a precursor to changes in VD. Additionally, comparisons should be taken in the context of the patient’s disease activity. Eyes that are quiet or become quiet on treatment may not experience SCP/DCP VD loss, whereas eyes that are active may have a decline. Most patients in our cohort (21/23 eyes) remained or became inactive by the end of the 2 years; further comparison to patients who remained active would be important.

In this study, we enrolled a sizable cohort of age-matched healthy participants, employing an approximately 2:1 ratio to ensure a robust basis for comparison. We utilized stringent image quality criteria in the data acquisition phases, improving the quality of the imaging data and enhancing reliability. Subsequent quantification of whole-image and extra-FAZ vessel densities using a standardized algorithm then provided comprehensive and comparable data. However, there were also limitations in our study. Accurate segmentation with projection artifacts led to exclusion of eyes with severe disease where anatomical layers were disrupted. Also, quantitative assessment of the choriocapillaris, including flow deficit analysis, was not performed.

Our study reveals microvascular changes using OCTA in a large sample of BCR-affected eyes. We demonstrate a decrease in macular SCP and DCP VD in BCR eyes compared with age-matched controls, and the association of VD with BCVA and disease duration. Our longitudinal study suggests overall that disease quiescence appears, at least in the short-term, to stabilize VD. Finally, our findings collectively suggest a potentially disproportionate and earlier impact on the DCP.
