# Supplementary material for: Characterization of Retinal Microvascular Abnormalities in Birdshot Chorioretinopathy Using OCT Angiography
Source: Ophthalmol Sci. 2024 Jun 17;4(6):100559. doi: 10.1016/j.xops.2024.100559 (PMC11334704; doi:10.1016/j.xops.2024.100559)
Supplement: Fig S5 [file mmc1.pdf]

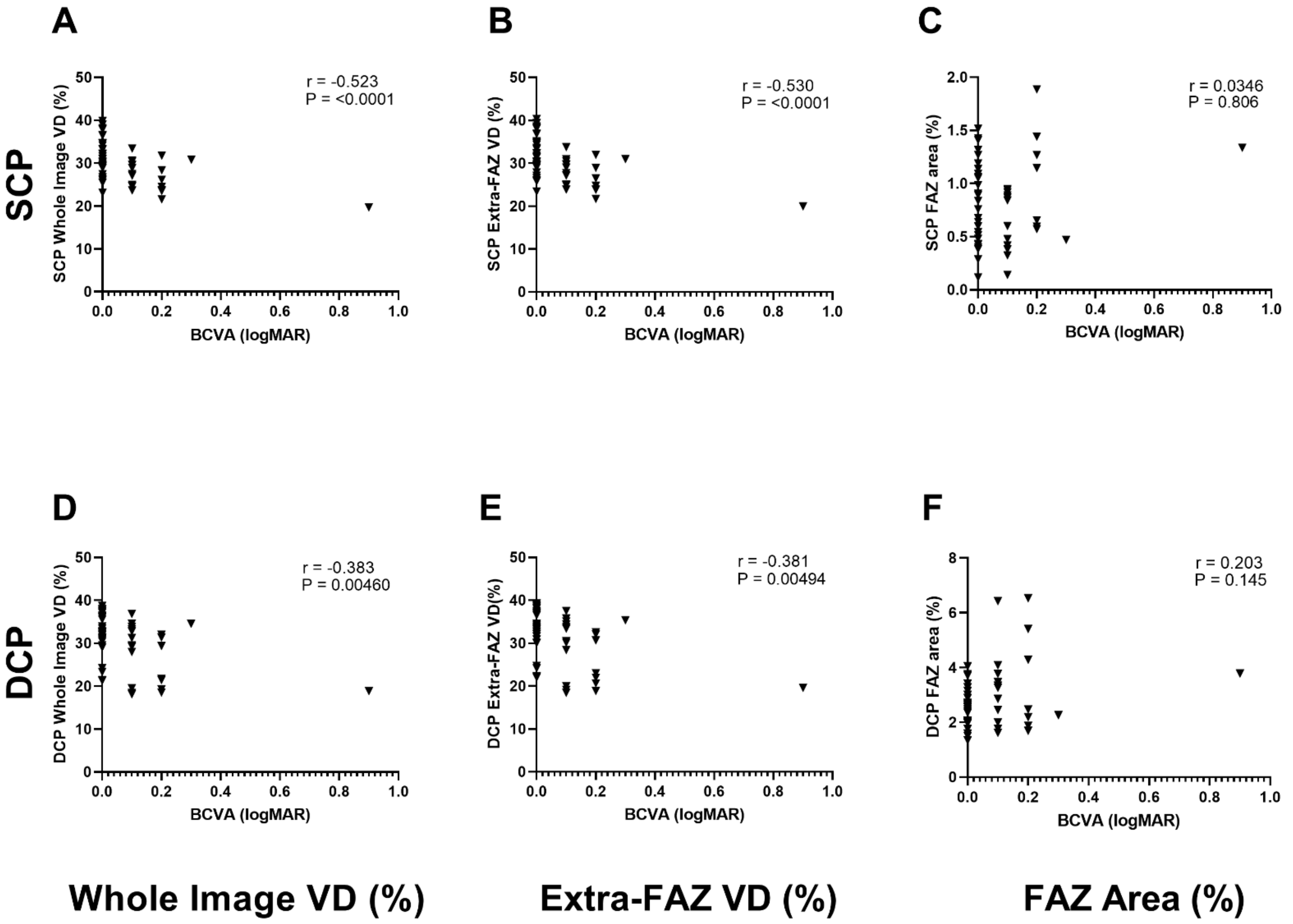

Figure S5. Scatter plot demonstrating the relationship of best corrected visual acuity (BCVA) with vessel analysis parameters: whole-image VD, extra-foveal avascular zone (extra-FAZ) VD, and foveal avascular zone (FAZ) area in the superficial capillary plexus (SCP, A – C) and the deep capillary plexus (DCP, D – F). VD = vessel density.
