# Supplementary material for: Characterization of Retinal Microvascular Abnormalities in Birdshot Chorioretinopathy Using OCT Angiography
Source: Ophthalmol Sci. 2024 Jun 17;4(6):100559. doi: 10.1016/j.xops.2024.100559 (PMC11334704; doi:10.1016/j.xops.2024.100559)
Supplement: Fig S6 [file mmc2.pdf]

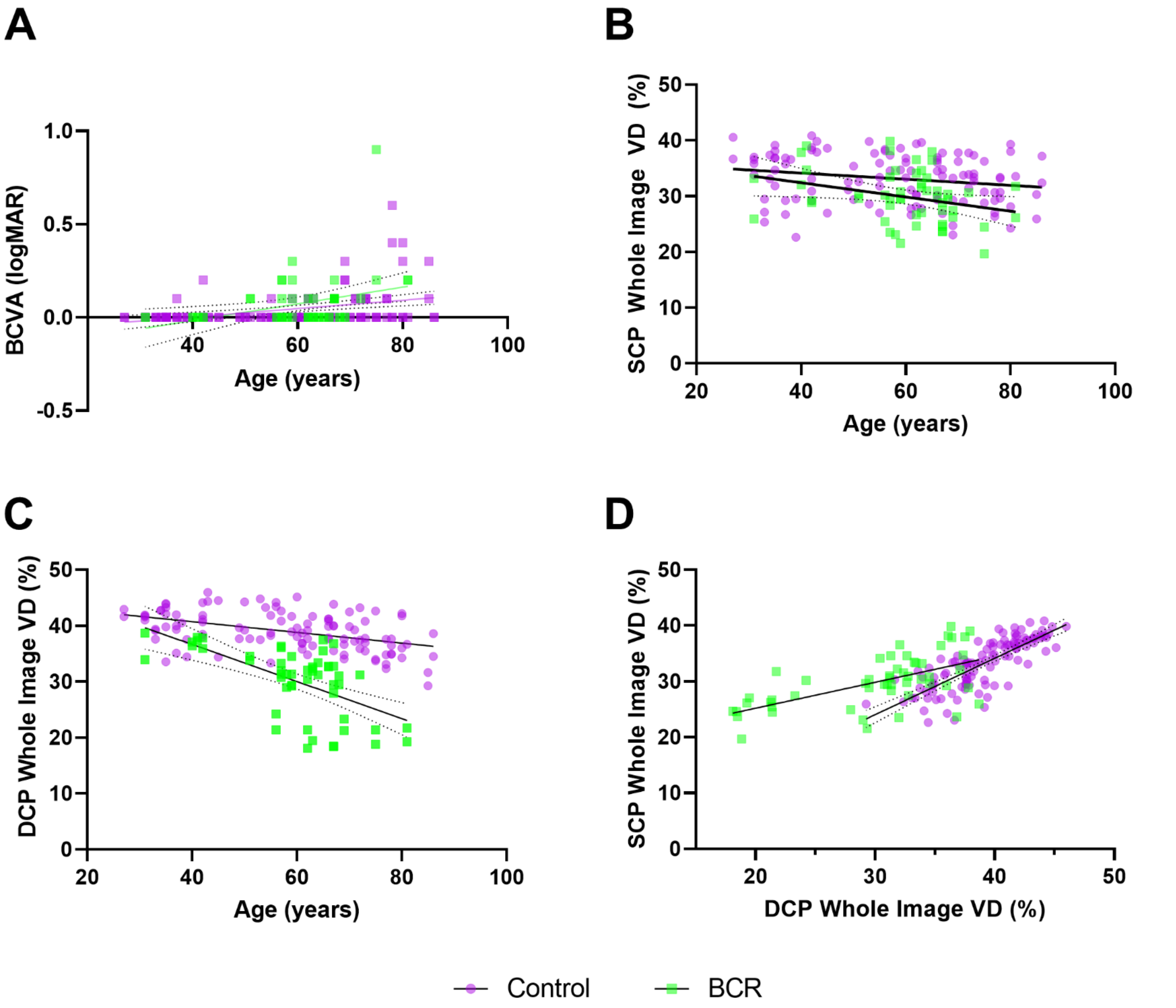

Figure S6. Comparing best corrected visual acuity (BCVA), superficial capillary plexus VD (SCP) and deep capillary plexus (DCP) VD between control and birdshot chorioretinitis (BCR) eyes. (A) Relationship between BCVA and age in control and BCR eyes. (B) Relationship between the SCP VD and age in control and BCR eyes. (C) Relationship between DCP VD and age in control and BCR eyes. (D) Relationship between SCP VD and DCP VD in control and BCR eyes. VD = vessel density.
