# Supplementary material for: Characterization of Retinal Microvascular Abnormalities in Birdshot Chorioretinopathy Using OCT Angiography
Source: Ophthalmol Sci. 2024 Jun 17;4(6):100559. doi: 10.1016/j.xops.2024.100559 (PMC11334704; doi:10.1016/j.xops.2024.100559)
Supplement: Table S6 [file mmc3.pdf]

Table S5. Results of multivariable analyses examining the relationship between baseline between baseline characteristics with each of the vessel analysis parameters.

Outcome	Covariate	Estimate	SE	95% CI Limits		*P*-value
				Minimum	Maximum	
SCP whole-image VD	Age (years)	-0.096	0.061	-0.22	0.023	0.11
	CST (µm)	0.0073	0.0067	-0.0059	0.021	0.28
	Disease duration (months)	-0.16	0.058	-0.27	-0.043	0.0069
	Treatment					
	Biologics	Reference	--	--	--	--
	No treatment	0.054	2.24	-4.34	4.45	0.98
	Oral treatment	2.09	2.45	-2.71	6.89	0.39
	Biol & oral treatment	1.59	2.64	-3.58	6.76	0.55
SCP extra-FAZ VD	Age (years)	-0.094	0.062	-0.21	0.027	0.13
	CST (µm)	0.0068	0.0067	-0.0063	0.020	0.31
	Disease duration (months)	-0.16	0.058	-0.28	-0.047	0.0055
	Treatment					
	Biologics	Reference	--	--	--	--
	No treatment	0.124	2.21	-4.20	4.45	0.96
	Oral treatment	2.20	2.43	-2.56	6.95	0.37
	Biol & oral treatment	1.68	2.61	-3.43	6.79	0.52
SCP FAZ Area	Age (years)	0.011	0.0039	0.0037	0.019	0.0038

	CST (µm)	-0.0020	0.0010	-0.0039	-0.00010	0.036
	Disease duration (months)	-0.0099	0.0062	-0.022	0.0022	0.11
	Treatment					
	Biologics	Reference	--	--	--	--
	No treatment	0.22	0.20	-0.16	0.61	0.25
	Oral treatment	0.26	0.21	-0.15	0.66	0.22
	Biol & oral treatment	0.25	0.23	-0.20	0.70	0.27
DCP extra-FAZ VD	Age (years)	-0.31	0.068	-0.44	-0.18	<0.0001
	CST (µm)	0.026	0.0060	0.014	0.038	<0.0001
	Disease duration (months)	-0.19	0.047	-0.28	-0.098	<0.0001
	Treatment					
	Biologics	Reference	--	--	--	--
	No treatment	-7.39	1.99	-11.30	-3.48	0.00020
	Oral treatment	-2.47	1.73	-5.87	0.93	0.15
	Biol & oral treatment	-3.60	2.01	-7.54	0.35	0.074
DCP whole-image VD	Age (years)	-0.31	0.065	-0.44	-0.19	<0.0001
	CST (µm)	0.027	0.0058	0.016	0.039	<0.0001
	Disease duration (months)	-0.18	0.044	-0.26	-0.091	<0.0001
	Treatment					
	Biologics	Reference	--	--	--	--
	No treatment	-7.42	1.95	-11.2	-3.59	0.00010

	Oral treatment	-2.55	1.71	-5.91	0.80	0.14
	Biol & oral treatment	-3.75	1.97	-7.62	0.11	0.057
DCP FAZ Area	Age (years)	0.050	0.020	0.012	0.089	0.011
	CST (µm)	-0.0053	0.0012	-0.0077	-0.0029	<0.0001
	Disease duration (months)	-0.026	0.015	-0.056	0.0039	0.089
	Treatment					
	Biologics	Reference	--	--	--	--
	No treatment	0.73	0.32	0.10	1.35	0.022
	Oral treatment	0.65	0.42	-0.17	1.46	0.12
	Biol & oral treatment	0.81	0.47	-0.11	1.72	0.085

CST = central subfield thickness; SCP = superficial capillary plexus; DCP = deep capillary plexus; FAZ = foveal avascular zone; VD = vessel density
